# Combined impact of multiple nutrients on longevity: findings from the Integrative Longevity Omics study

**DOI:** 10.1093/geroni/igaf122.4409

**Published:** 2025-12-31

**Authors:** Paola Sebastiani, Andres Ardisson Korat, Anastasia Leshchyk, Nicole Roth, Toshiko Tanaka, Stacy Andersen, Thomas Perls

**Affiliations:** Tufts Medical Center, Boston, Massachusetts, United States; Tufts University, Boston, Massachusetts, United States; Tufts Medical Center, Boston, Massachusetts, United States; Boston University, Boston, Massachusetts, United States; National Institute on Aging, Baltimore, Maryland, United States; Boston University, Boston, Massachusetts, United States; Boston University, Boston, Massachusetts, United States

## Abstract

Understanding the effect of nutrition on aging is essential for identifying dietary habits that promote healthy longevity. Many studies have investigated the effect of nutrient patterns on aging trajectories by analyzing nutrients in isolation or aggregated into dietary indices. To consider the joint effects of multiple nutrient groups simultaneously while accounting for their interactions, we developed the Nutrient Variety Index (NVI) that summarizes the distribution of groups of nutrients within a diet. We generated NVIs of 19 nutrient groups from dietary data of 235 centenarians and 469 centenarians’ offspring and controls enrolled in the Integrative Longevity Omics study, 8035 Health and Retirement Study participants, and 1500 Baltimore Longitudinal Study of Aging participants. We analyzed NVIs in relation to chronological and biological age, cognitive function, mortality risk, and exceptional longevity. Our analyses showed substantial changes of the NVIs of multiple nutrient groups with older ages. For example, the NVI of proportion of carbohydrates, fats, proteins and alcohol in the diet was negatively correlated with older age and mortality risk and positively correlated with younger biological age based on various metabolomic clocks. The decreasing NVI of these macronutrients with older age captures the shift in balance between carbohydrates that become the dominant nutrients in the diet of individuals aged 60 years and older, compared to decreased consumption of fats and proteins. Individuals selected for familial extreme longevity had a significantly higher NVI of these and other nutrients groups, thus suggesting that they maintain a better balance of many nutrients as they age.

